# Telmisartan and Rosuvastatin Synergistically Ameliorate Dementia and Cognitive Impairment in Older Hypertensive Patients With Apolipoprotein E Genotype

**DOI:** 10.3389/fnagi.2020.00154

**Published:** 2020-06-09

**Authors:** Wenjing Hu, Ying Li, Yingxin Zhao, Yuanli Dong, Yi Cui, Shangwen Sun, Gary Gong, Hua Zhang, Qiang Chai, Juan Wang, Zhendong Liu

**Affiliations:** ^1^Institute of Basic Medicine, Shandong Provincial Hospital Affiliated to Shandong First Medical University, Jinan, China; ^2^School of Medicine and Life Sciences, University of Jinan-Shandong Academy of Medical Sciences, Jinan, China; ^3^Department of Community, Lanshan District People Hospital, Linyi, China; ^4^Department of Radiology, Qilu Hospital of Shandong University, Jinan, China; ^5^The Russel H. Morgan Department of Radiology and Radiological Sciences, The Johns Hopkins University School of Medicine, Baltimore, MD, United States; ^6^Department of Cardiology, The Second Hospital of Shandong University, Jinan, China

**Keywords:** dementia, cognitive impairment, apolipoprotein E, lipid lowering, anti-hypertension, hypertension

## Abstract

**Objective**: To investigate the effect of telmisartan, rosuvastatin, or their combination on dementia and to understand the impact of *apolipoprotein E* (*APOE*) genotype on the effect of the medications in older patients with hypertension.

**Methods**: This is a double-blind, randomized, and placebo-controlled trial using a 2 × 2 factorial design. Between April 2008 and November 2010, 1,244 hypertensive patients aged ≥60 years without cognitive impairment were recruited from communities in six cities in Shandong area, China. Patients were randomized into telmisartan and rosuvastatin administration after a 2-week washout period. *APOE* genotype was identified at the baseline. Possible dementia was determined using the combination of the global cognitive function and Assessment of the Informant Questionnaire on Cognitive Decline in the Elderly (IQCODE).

**Results**: Over an average follow-up of 7 [interquartile range (IQR): 6.7–7.2] years, telmisartan and rosuvastatin significantly reduced the cognitive impairment progression and the incidence of dementia. There was a synergistic interaction between telmisartan and rosuvastatin to reduce the cognitive impairment and the incidence of dementia (*P*_adjusted_ < 0.001). The cognitive impairment progression and the risk of dementia were higher in the hypertensive patients with *APOE ε4* allele than in those without *APOE ε4* allele. Rosuvastatin medication significantly alleviated the cognitive impairment progression and the risks of dementia in patients with *APOE ε4* allele.

**Conclusion**: The combination of telmisartan and rosuvastatin might be an effective prevention and/or treatment strategy for cognitive impairment and dementia, especially in hypertensive patients with the *APOE ε4* allele.

**Clinical Trial Registration:** www.ClinicalTrials.gov, ChiCTR.org.cn, identifier ChiCTR-IOR-17013557. Registered on April 12, 2017 – Retrospectively registered, http://www.chictr.org.cn/showproj.aspx?proj=23121

## Introduction

Dementia is an age-related and progressive neurodegenerative disease characterized by a decline in cognitive function that affects the quality of life in older adults (Peng et al., 2014; Sabia et al., 2017). Epidemiological studies reported 47 million cases of dementia worldwide in 2015 and have predicted that this will triple by 2050, given the steadily increasing life expectancy (Prince et al., 2013; Sabia et al., 2017), placing a heavy burden on individuals, families, and society. Hence, preventing and delaying the progression of dementia and cognitive impairment is a global public health issue (Shah et al., 2016).

Dementia and cognitive impairment are multifactorial diseases that are most frequently caused by aging, hypertension, dyslipidemia, and genetic factors (Farao and Iadecola, 2013; Sabia et al., 2017; Van Middelaar et al., 2018). Although hypertension is often accompanied by dyslipidemia, it is debatable whether antihypertensive treatment can improve cognitive outcome in the elderly (Farao and Iadecola, 2013; Sörös et al., 2013), and there are no specific classes of antihypertensive agents that have consistently shown greater efficacy in alleviating dementia or cognitive impairment (Gorelick et al., 2011; Farao and Iadecola, 2013). Moreover, it is unclear whether lipid-lowering statin treatment is beneficial, since different studies have reported conflicting findings (Jick et al., 2000; Hajjar et al., 2002; Rodrigues et al., 2002).

Sartans are some of the widely used antihypertensive medications. This popularity of sartans is due to its superior efficacy and tolerability profiles and the long duration of action. Sartans have been demonstrated to protect against cognitive deficits in animal models of Alzheimer’s disease (Kehoe et al., 2009; Ahmed et al., 2018) and in human beings (Petek et al., 2018). However, there is an argument on the beneficial effect of sartans on cognitive decline (Diener et al., 2008; Tsukuda et al., 2009; Ho and Nation, 2017).

Statins has been recommended by guidelines as the medication for primary prevention of cardiovascular disease (Bibbins-Domingo et al., 2016; Mortensen and Nordestgaard, 2018; Arnett et al., 2019). A recent meta-analysis study demonstrated that statin medications could produce significant reductions in major vascular events irrespective of age (Cholesterol Treatment Trialists’ Collaboration, 2019). Studies also showed that statins possess beneficial effects on reducing stroke risks and ameliorating dementia (Power et al., 2015; Geifman et al., 2017; Zissimopoulos et al., 2017; Petek et al., 2018). However, similar to sartans, the beneficial effect of statins on cognitive decline also remains controversial (Power et al., 2015; Geifman et al., 2017; Zissimopoulos et al., 2017; Sinyavskaya et al., 2018).

Several genes have been identified as risk factors for dementia and cognitive impairment in genome-wide association studies (Dergunov, 2011; Nazarian et al., 2019). One of these, *apolipoprotein E* (*APOE*), has three common alleles (*ε2*, *ε3*, and ε4) encoding distinct protein isoforms (Dergunov, 2011; Ji et al., 2018). In particular, the *APOE* ε4 allele is closely associated with elevated levels of total cholesterol and low-density lipoprotein cholesterol (Dergunov, 2011) with increased risk of dementia and cognitive impairment (Mahely and Huang, 1999; Reitz, 2012). However, the relationships between the *APOE* gene and blood pressure and *APOE* gene and plasma lipid-lowering treatments are not well understood. To address this issue, the present study investigated how the *APOE* ε4 genotype influences the effects of sartans and statins on dementia and cognitive impairment risk in older hypertensive patients.

## Methods

### Standard Protocol Approval, Registration, and Patient Consent

This study was conducted in compliance with the Declaration of Helsinki and adhered to good clinical practice guidelines. The study protocol was approved by the Research Ethics Committee of the Institute of Basic Medicine, Shandong Academy of Medical Sciences, and was retrospectively registered with ChiCTR.org.cn (ChiCTR-IOR-17013557). Written informed consents were obtained from all the patients.

### Study Design and Sample Size Determination

This study was a double-blind, randomized, and placebo-controlled trial using a 2 × 2 factorial design. The protocol of this study is described elsewhere (Ji et al., 2018). The major objective was to investigate the interaction of sartans and low-dose statins on the incident cardio- and cerebro-vascular events including stroke and myocardial infarction. The sample size was determined using the stroke incidence, and 1,244 essential hypertensive elderly aged ≥60 years were determined (Ji et al., 2018). In this study, we mainly investigated the interaction of sartans, statins, and *APOE* genotype on dementia incidence and the trajectory of global cognitive function during the study period. So, the sample size should be determined using the incidence of dementia. Given that the incidence of dementia is 5.1% per year (Jia et al., 2014), the sample size of 1,244 patients could well provide a statistical power of 90% including taking into account a dropout rate of 10% in this study.

### Study Patients

Details of the study patients are as described in our previous study (Ji et al., 2018). Briefly, 1,244 essential hypertensive patients aged ≥60 years were eligible and recruited from communities in six cities in Shandong, China, between April 2008 and November 2010. The exclusion criteria were patients with Alzheimer’s disease, Parkinson’s disease, schizophrenia, seizures, Mini-Mental State Examination (MMSE) score ≤23, secondary hypertension, diabetes mellitus, myocardial infarction or stroke in the last 6 months, clear hypersensitivity or contraindication to the medications administered in the study, chronic liver disease or renal dysfunction, inflammatory muscle disease, connective tissue diseases or malignancy, drug or alcohol abuse, intention to leave current residence within 6 years, inability to walk to the clinic, and unwillingness to provide informed consent.

### Study Randomization, Intervention, and Follow-Up

After a 2-week washout period, the patients were randomized on a 1:1:1:1 ratio into control (telmisartan placebo and rosuvastatin placebo), telmisartan (telmisartan activator and rosuvastatin placebo), rosuvastatin (telmisartan placebo and rosuvastatin activator), and combination (telmisartan activator and rosuvastatin activator) groups. A computer-generated randomization was conducted by members who were not directly working on the study according to the order of recruitment with a block size of eight without stratification. Each patient was assigned a unique number that was used throughout the study. The investigators and patients were masked to treatment assignment until the completion of the study and until final clinical database lockdown.

Telmisartan was administered at a concentration of either 40 mg or increased to 80 mg once daily if needed and rosuvastatin at 10 mg once daily. Hydrochlorothiazide (12.5 mg increased to 25 mg once daily if needed) was used as an open-label medication and a background treatment in the four groups. Patients were visited weekly during the washout period, then at trial months 1, 3, and 6, every 6 months thereafter, and at final visit.

### Evaluation of Global Cognitive Function

Assessments of global cognitive function include the MMSE (Liu et al., 2016; Duan et al., 2017), Montreal Cognitive Assessment (MoCA; Duan et al., 2017), Mattis Dementia Rating Scale (DRS; Chan et al., 2001), and Clinical Dementia Rating (CDR; Yue et al., 2016). The scales were implemented at baseline, annual follow-up, and final visits using the Chinese versions. All tests were conducted by experienced neuropsychology research assistants who were blinded to clinical and laboratory data, genotype, and psychological outcomes. The MMSE, MoCA, DRS, and CDR are widely used standard tests for assessing, screening, and staging cognitive dysfunction and dementia with excellent test–retest and inter-rater reliability. Lower MMSE, MoCA, and DRS scores reflect more severe cognitive impairment and dementia, whereas a higher CDR score represents more severe dementia. After testing 90 patients in random, the coefficients of variation of the interobserver were 0.91 for the MMSE score, 0.89 for the MoCA score, 0.87 for DRS score, and 0.84 for CDR score.

### The Informant Questionnaire on Cognitive Decline in the Elderly

Assessment of the Informant Questionnaire on Cognitive Decline in the Elderly (IQCODE) was implemented at baseline, biennial follow-up, and final visits. The IQCODE is one of the worldwide used informant (proxy)-rated complementary screening tool for dementia that rates the change in cognitive function from a previous level of 10 years earlier (Jorm and Jacomb, 1989; Jorm, 1994, 2004). It is available in various versions (Jorm and Jacomb, 1989; Jorm, 1994, 2004; Harrison et al., 2016), has demonstrated utility in multiple cultural groups (Jorm and Jacomb, 1989; Fuh et al., 1995; Morales et al., 1995; Harrison et al., 2016), and has high internal (alpha = 0.95) and test–retest (correlation coefficient = 0.75) reliabilities (Jorm and Jacomb, 1989; Gavett et al., 2011). Our study used a shortened version consisting of 16 items, which was demonstrated essentially to be comparable to the original version (Jorm, 1994), to measuring cognitive decline during the follow-up period. The IQCODE was independently completed by the spouses, relatives, friends, or carers who closely knew the patients for at least 10 years. Each item on the IQCODE is rated on a five-point scale, where 1 represents “Much better,” 2 “A bit better,” 3 “Not much change,” 4 “A bit worse,” and 5 “Much worse.” The final score is the average of the rated 16 item scores. A higher score represents a greater cognitive impairment. The coefficient of variation of the interobserver was 0.93 after being tested in random samples of 90 patients.

### *APOE* Genotyping

DNA was extracted from 10 ml peripheral blood mixed with ethylenediaminetetraacetic acid (EDTA). *APOE* genotyping based on the presence of the single-nucleotide polymorphisms rs429358 and rs7412 was carried out by polymerase chain reaction using the TaqMan genotyping kit (Applied Biosystems, Foster City, CA, USA). The primers used were forward primer: 5′-TTG AAG GCC TAC AAA TCG GAA CTG-3′ and reversed primer: 5′-CCG GCT GCC CAT CTC CTC CAT CCG-3′ (Molinuevo et al., 2016). Patients were categorized as *ε4*-positive carriers (genotypes *ε*2/ε4, ε3/ε4, or ε4/ε4) or ε4-negative carriers (genotypes ε2/ε2, ε2/ε3, or ε3/ε3; Ji et al., 2018).

### Outcomes

Primary outcomes included changes in global cognitive function including MMSE, MoCA, DRS, and CDR scores and dementia incidence. Possible dementia was diagnosed depending on a combination of IQCODE and global cognitive function assessment according to the recommendations from the National Institute on Aging–Alzheimer’s Association workgroups on diagnostic guidelines for Alzheimer’s disease (Narasimhalu et al., 2008; Albert et al., 2011). The cut-off of MMSE was ≤23 points or a decline of ≥3 points between any two annual follow-up visits (Liu et al., 2016; Duan et al., 2017), MoCA was >20 points (Delgado et al., 2017), DRS was >120 points (Chan et al., 2001), CDR was ≥1.0 (Yue et al., 2016), and IQCODE was ≥3.38 (Biessels et al., 2006). The secondary outcomes were incident stroke and all-cause mortality during the follow-up period.

### Statistical Analysis

The intention-to-treat principle was followed in the analysis of this study. Continuous data are expressed as mean with standard deviation (SD) or the median with interquartile range (IQR; the range between the 25th and 75th percentiles) depending on the normality of the data, and categorical data are expressed as a frequency with percentages. The Kolmogorov–Smirnov test was used to assess the normality of the continuous data. Comparisons of continuous data between groups were performed with the one-way analysis of variance (ANOVA) with the Bonferroni procedure or Kruskal–Wallis H test with a Wilcoxon rank-sum test depending on the normality of the data; categorical data were compared with the chi-square test. A linear mixed model was used to assess differences in the trajectory of MMSE, MoCA, CDR, and DRS scores over the follow-up period among groups. A Kaplan–Meier analysis with the log-rank test was used to evaluate differences in the risks of dementia incidence. The Cox proportional hazards model was used to assess the hazard ratio (HR) and 95% confidence interval (CI).

In this study, we tested the interactions between telmisartan and rosuvastatin; *APOE ε4* allele status and telmisartan; *APOE ε4* allele status and rosuvastatin; and among *APOE ε4* allele status, telmisartan, and rosuvastatin on the trajectory of global cognitive function and the dementia incidence. First, we classified patients into control, telmisartan, rosuvastatin, and combination groups to investigate the interaction between telmisartan and rosuvastatin. Then, we reclassified patients into telmisartan placebo * ε4 negative/positive [telmisartan(−) * ε4(−)/(+)] and telmisartan activator * ε4 negative/positive [telmisartan(+) * ε4(−)/(+)] groups to investigate the interaction between telmisartan and *APOE ε4* allele status. Third, we reclassified patients into rosuvastatin placebo * ε4 negative/positive [rosuvastatin(−) * ε4(−)/(+)] and rosuvastatin activator * ε4 negative/positive [rosuvastatin(+) * ε4(−)/(+)] groups to investigate the interaction between rosuvastatin and *APOE ε4* allele status. Finally, we subclassified the control, telmisartan, rosuvastatin, and combination groups into control * ε4(−)/(+), telmisartan * ε4(−)/(+), rosuvastatin * ε4(−)/(+), and combination * ε4(−)/(+) groups, respectively, to investigate the interaction among *APOE ε4* allele status, telmisartan, and rosuvastatin.

Models were adjusted for age, sex, education, smoking, alcohol consumption, baseline body mass index, baseline blood pressure, baseline fasting plasma glucose, baseline plasma lipids, the status of hydrochlorothiazide administration, changes in blood pressure, lipids, and fasting plasma glucose during the trial period, and the stroke incidence during the trial period. Multiple sensitivity analyses were performed using: (1) first diagnosed dementia during the trial period; (2) multiple imputation by chained equations for imputing missing data for variables; (3) stratified analysis to make sure that the associations found are robust; and (4) confounders included the changes in blood pressure, lipids, and fasting plasma glucose and the stroke incidence during the trial period in models. Statistical analyses were performed using SPSS v.24.0 (SPSS Inc., Chicago, IL, USA). A two-sided *P*-value < 0.05 was considered statistically significant.

## Results

### Baseline Characteristics

**Figure 1:**
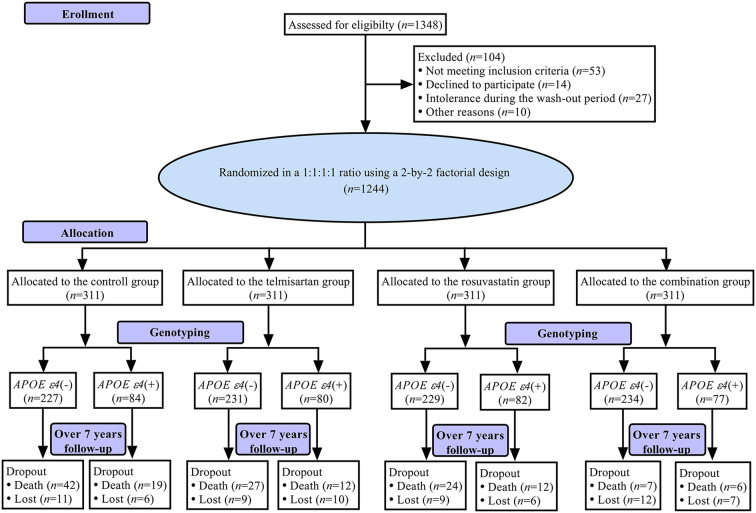
Flowchart.

Figure 1 shows a flowchart of this study. The first patient was recruited on April 28, 2008, and the final follow-up visit was completed on September 25, 2017. The average follow-up period was 7.0 (IQR: 6.7–7.2) years. The mean age at baseline was 70.11 ± 6.08 years, 597 (48.0%) subjects were female, and 323 (26.0%) were *APOE ε4*(+). The demographic and baseline clinical characteristics of the patients in control, telmisartan, rosuvastatin, and combination groups are shown in Table 1. There were no significant differences in the demographic and clinical characteristics among the four groups (all *P* > 0.05).

**Table 1:** Demographic and clinical characteristics of the study population at baseline.

	Control group	Telmisartan group	Rosuvastatin group	Combination group	*P*-value
	(*n* = 311)	(*n* = 311)	(*n* = 311)	(*n* = 311)	
Age (years)	70.12 ± 6.17	70.48 ± 6.20	70.15 ± 5.96	69.68 ± 6.00	0.437
Female [*n* (%)]	147 (47.3)	151 (48.6)	155 (49.8)	144 (46.3)	0.829
Education (years)	7.00 (5.00, 10.00)	7.00 (3.00, 10.00)	7.00 (5.00, 10.00)	7.00 (4.00, 10.00)	0.416
Smoking [*n* (%)]	84 (27.0)	79 (25.4)	72 (23.2)	79 (25.4)	0.665
Alcohol consumption [*n* (%)]	110 (35.4)	120 (38.6)	108 (34.7)	107 (34.4)	0.684
BMI (kg/m^2^)	24.49 ± 3.08	24.78 ± 2.89	24.55 ± 2.62	25.01 ± 3.77	0.144
SBP (mm Hg)	156.73 ± 9.99	156.91 ± 9.83	156.16 ± 9.29	156.67 ± 10.11	0.801
DBP (mm Hg)	70.80 ± 7.83	71.13 ± 7.23	71.36 ± 7.20	70.84 ± 7.63	0.767
Total cholesterol (mmol/L)	5.08 ± 0.63	5.08 ± 0.60	5.05 ± 0.66	5.00 ± 0.60	0.416
Triglycerides (mmol/L)	1.50 ± 0.36	1.51 ± 0.38	1.45 ± 0.36	1.50 ± 0.38	0.177
HDL-c (mmol/L)	1.20 ± 0.21	1.18 ± 0.22	1.20 ± 0.21	1.19 ± 0.19	0.593
LDL-c (mmol/L)	3.19 ± 0.62	3.21 ± 0.63	3.19 ± 0.68	3.13 ± 0.63	0.396
FPG (mmol/L)	5.46 ± 0.78	5.46 ± 0.81	5.42 ± 0.74	5.48 ± 0.75	0.806
MMSE (score)	29.00 (28.00, 30.00)	29.00 (28.00, 30.00)	29.00 (28.00, 30.00)	29.00 (28.00, 30.00)	0.823
MoCA (score)	28.00 (27.00, 29.00)	28.00 (27.00, 29.00)	28.00 (27.00, 29.00)	28.00 (27.00, 29.00)	0.275
DRS (score)	135.00 (128.00, 139.00)	134.00 (129.00, 138.00)	134.00 (129.00, 139.00)	134.00 (129.00, 139.00)	0.950
CDR (score)	0.00 (0.00, 0.00)	0.00 (0.00, 0.00)	0.00 (0.00, 0.00)	0.00 (0.00, 0.00)	1.00
IQCODE (score)	1.79 (1.27, 2.35)	1.81 (1.26, 2.29)	1.73 (1.3, 2.28)	1.78 (1.34, 2.26)	0.974
*APOE ε4*(+) [n (%)]	84 (27.0)	80 (25.7)	82 (26.4)	77 (24.8)	0.930

*Data are shown as *n* (percentage), mean ± standard deviation, or median (interquartile range).* Abbreviations*: BMI, body mass index; SBP, systolic blood pressure; DBP, diastolic blood pressure; HDL-c, high-density lipoprotein cholesterol; LDL-c, low-density lipoprotein cholesterol; FPG, fasting plasma glucose; MMSE, Mini-Mental State Examination; MoCA, Montreal Cognitive Assessment; DRS, Mattis Dementia Rating Scale; CDR, Clinical Dementia Rating; IQCODE, Informant Questionnaire on Cognitive Decline in the Elderly; APOE, apolipoprotein E*.

### Outcomes

Over the follow-up period, a total of 176 patients (2.0% per year) developed dementia. The scores of MMSE, MoCA, and DRS were declined, and the scores of CDR and IQCODE were increased relative to the baseline.

### Interactions Between Telmisartan and Rosuvastatin

**Figure 2:**
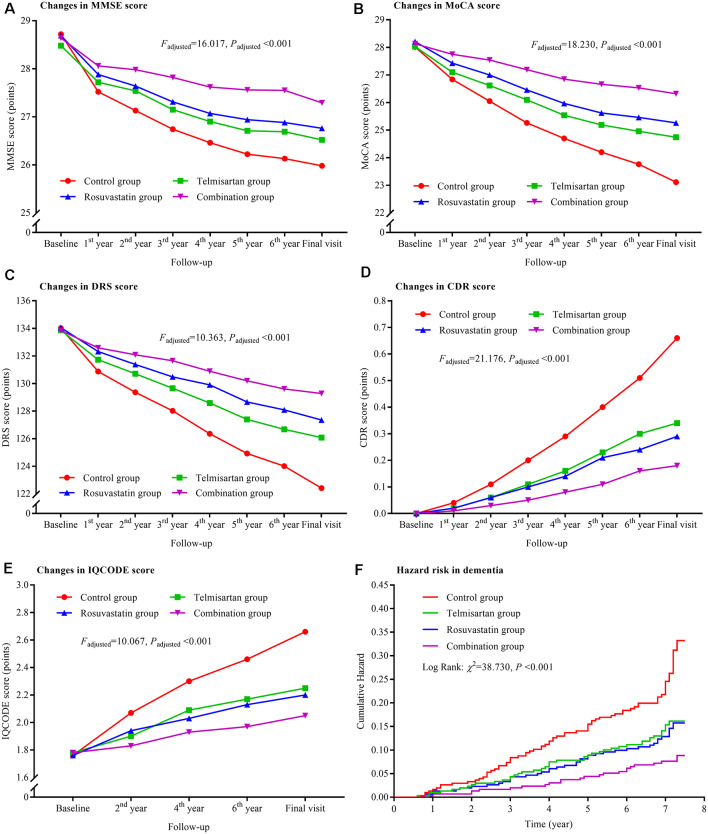
Trajectory of global cognitive function and IQCODE and cumulative hazards of dementia incidence during the follow-up period in the patients grouped by telmisartan and rosuvastatin administration. Panel **(A)** is the trajectory of MMSE; panel **(B)** is the trajectory of MoCA; panel **(C)** is the trajectory of DRS; panel **(D)** is the trajectory of CDR; panel **(E)** is the trajectory of IQCODE, and panel **(F)** is the cumulative hazard of dementia incidence. MMSE, Mini-Mental Scale Estimation; MoCA, Montreal Cognitive Assessment; DRS, Mattis Dementia Rating Scale; CDR, Clinical Dementia Rating; IQCODE, Informant Questionnaire on Cognitive Decline in the Elderly.

The trajectories of MMSE, MoCA, DRS, CDR, and IQCODE scores in the duration of follow-up are presented in Figure 2. There were declining trends in MMSE, MoCA, and DRS and increasing trends in CDR and IQCODE in the four groups. However, the differences in the trends were significant among the four groups after adjustment for confounders as above described (all *P*_adjusted_ < 0.001). The declining trends in MMSE, MoCA, and DRS and the increasing trends in CDR and IQCODE were significantly lower in the combination group than in the control, telmisartan, and rosuvastatin groups (all *P*_adjusted_ < 0.05). These trends in telmisartan and rosuvastatin groups were slower in the telmisartan and rosuvastatin groups when compared with the control group (all *P*_adjusted_ < 0.05). Moreover, similar results were found when we further compared the changes in MMSE, MoCA, DRS, CDR, and IQCODE scores from the baseline among the four groups (all *P*_adjusted_ < 0.05; Supplementary Figure S1). There were significant differences in the incidences of dementia among the four groups after adjustment for covariates (*P*_adjusted_ < 0.001). The risks of dementia in the combination, telmisartan, and rosuvastatin groups were significantly lower when compared to the control group (all *P*_adjusted_ < 0.001; Figure 2, Supplementary Table S1). Compared with the combination group, the risks of dementia were higher in the telmisartan group and the rosuvastatin group (all *P*_adjusted_ < 0.05). There was a significant interaction between telmisartan and rosuvastatin based on the changing trends in MMSE (*P*_adjusted_ = 0.011), MoCA (*P*_adjusted_ = 0.007), DRS (*P*_adjusted_ = 0.013), CDR (*P*_adjusted_ = 0.019), and IQCODE (*P*_adjusted_ = 0.014), and the incidences of dementia (*P*_adjusted_ < 0.001) after adjustment for confounders including the changes in blood pressure and lipids and the stroke incidence during the trial period.

### Interactions Between Telmisartan and *APOE ε4* Allele

**Figure 3:**
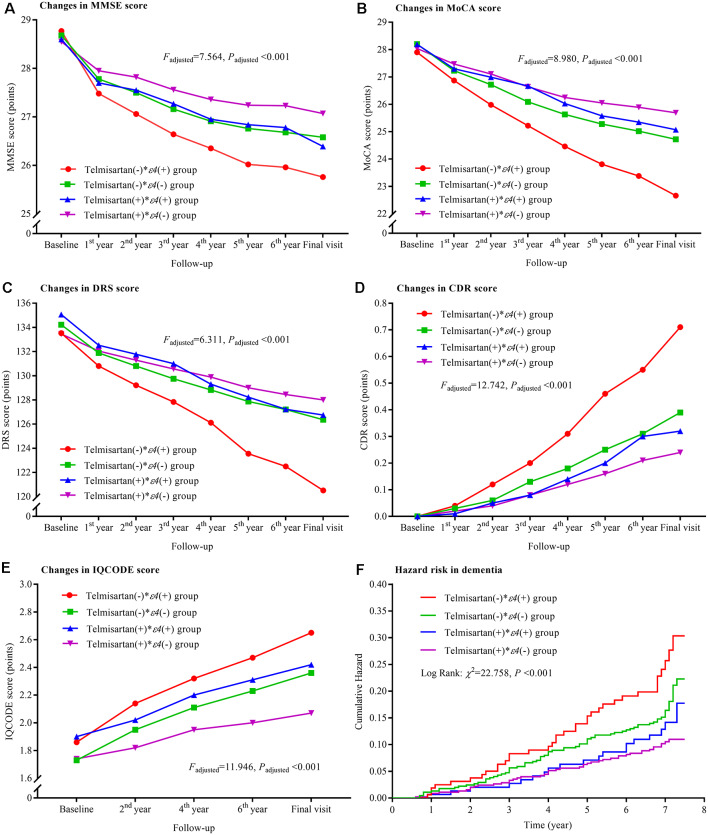
Trajectory of global cognitive function and IQCODE and cumulative hazards of dementia incidence during the follow-up period in the patients grouped by telmisartan administration and *APOE* genotype. Panel **(A)** is the trajectory of MMSE; panel **(B)** is the trajectory of MoCA; panel **(C)** is the trajectory of DRS; panel **(D)** is the trajectory of CDR; panel **(E)** is the trajectory of IQCODE, and panel **(F)** is the cumulative hazard of dementia incidence. MMSE, Mini-Mental Scale Estimation; MoCA, Montreal Cognitive Assessment; DRS, Mattis Dementia Rating Scale; CDR, Clinical Dementia Rating; IQCODE, Informant Questionnaire on Cognitive Decline in the Elderly; APOE, apolipoprotein E.

Compared to the telmisartan(−) * ε4(+) group, the declining trends in MMSE, MoCA, and DRS and the increasing trends in CDR and IQCODE were significantly lower in the other three groups after adjustment for confounders (all *P*_adjusted_ < 0.05; Figure 3). The declining trends in MMSE and MoCA in the telmisartan(+) * ε4(−) group were lower than those in the telmisartan(−) * ε4(−) group (*P*_adjusted_ = 0.002 and = 0.023, respectively). The increasing trend in IQCODE in the telmisartan(−) * ε4(−) group was lower than that in the telmisartan(−) * ε4(+) group (*P*_adjusted_ = 0.017). We further compared the changes in MMSE, MoCA, DRS, CDR, and IQCODE scores from the baseline among the four groups (Supplementary Figure S2). The changes in MMSE, MoCA, DRS, CDR, and IQCODE scores from the baseline were significantly higher in the telmisartan(−) * ε4(+) group than the other three groups (all *P*_adjusted_ < 0.05). The risks of dementia in the telmisartan(+) * ε4(−) group were lower when compared to the telmisartan(−) * ε4(−) and telmisartan(−) * ε4(+) groups (Figure 3, Supplementary Table S1). No statistical interaction was observed between telmisartan and *APOE ε4* allele on the changes in MMSE, MoCA, DRS, CDR, and IQCODE and in the incidences of dementia (all *P*_adjusted_ > 0.05).

### Interactions Between Rosuvastatin and *APOE ε4* Allele

**Figure 4:**
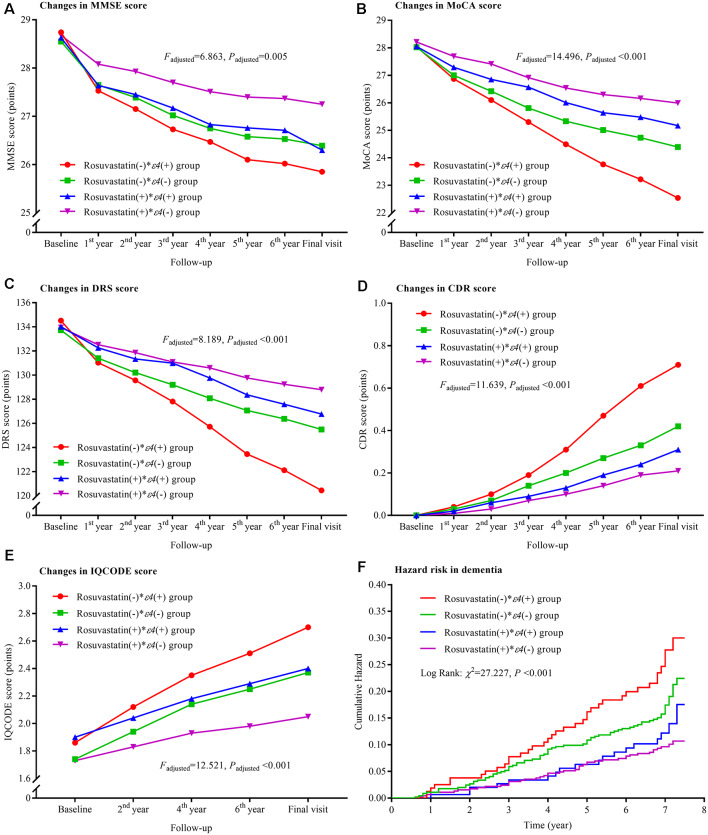
Trajectory of global cognitive function and IQCODE and cumulative hazards of dementia incidence during the follow-up period in the patients grouped by rosuvastatin administration and *APOE* genotype. Panel **(A)** is the trajectory of MMSE; panel **(B)** is the trajectory of MoCA; panel **(C)** is the trajectory of DRS; panel **(D)** is the trajectory of CDR; panel **(E)** is the trajectory of IQCODE, and panel **(F)** is the cumulative hazard of dementia incidence. MMSE, Mini-Mental Scale Estimation; MoCA, Montreal Cognitive Assessment; DRS, Mattis Dementia Rating Scale; CDR, Clinical Dementia Rating; IQCODE, Informant Questionnaire on Cognitive Decline in the Elderly; APOE, apolipoprotein E.

The declining trends in MMSE, MoCA, and DRS and the increasing trends in CDR and IQCODE and the risks of the dementia were significantly lower in the rosuvastatin(+) * ε4(−) group than in the rosuvastatin (−) * ε4(+) and rosuvastatin(−) * ε4(−) groups (all *P*_adjusted_ < 0.05; Figure 4). The declining trends in MoCA and DRS and the increasing trend in CDR were lower in the rosuvastatin(+) * ε4(+) group than in the rosuvastatin(−) * ε4(+) group (all *P*_adjusted_ < 0.05). The increasing trend in IQCODE was lower in the rosuvastatin(+) * ε4(−) group than in the rosuvastatin(−) * ε4(+) and lower in the rosuvastatin(−) * ε4(−) group than in the rosuvastatin(−) * ε4(+) group (all *P*_adjusted_ < 0.05). We further found that there were significant differences in the changes in MMSE, MoCA, DRS, CDR, and IQCODE scores from the baseline among the four groups (all *P*_adjusted_ < 0.05; Supplementary Figure S3). However, the differences in the changes in MMSE, MoCA, DRS, CDR, and IQCODE scores from the baseline were not significant between rosuvastatin(+) * ε4(+) and rosuvastatin(+) * ε4(−) group (all *P*_adjusted_ > 0.05). The risks of the dementia incidence were significantly lower in the rosuvastatin(+) * ε4(−) group than in the rosuvastatin(−) * ε4(+) and rosuvastatin(−) * ε4(−) groups (all *P*_adjusted_ < 0.05; Figure 4, Supplementary Table S1). The statistical interactions were observed between rosuvastatin and *APOE ε4* allele on the changes in MMSE (*P*_adjusted_ = 0.018), MoCA (*P*_adjusted_ = 0.020), DRS (*P*_adjusted_ = 0.031), CDR (*P*_adjusted_ = 0.027), and IQCODE (*P*_adjusted_ = 0.022) and in the dementia (*P*_adjusted_ = 0.022).

### Interactions Among Telmisartan, Rosuvastatin, and *APOE ε4* Allele

**Figure 5:**
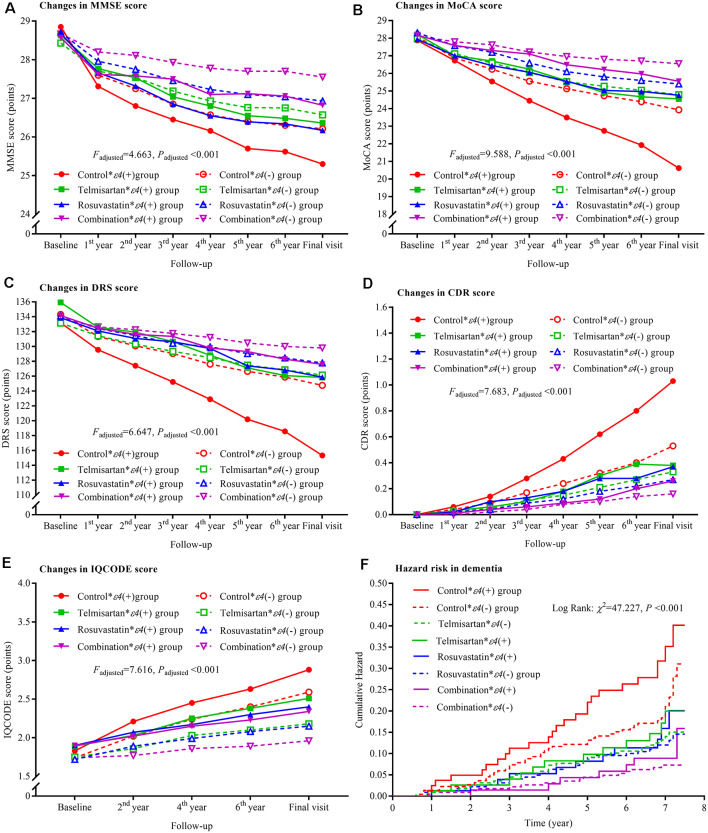
Trajectory of global cognitive function and IQCODE and cumulative hazards of dementia incidence during the follow-up period in the patients grouped by telmisartan, rosuvastatin, and *APOE* genotype. Panel **(A)** is the trajectory of MMSE; panel **(B)** is the trajectory of MoCA; panel **(C)** is the trajectory of DRS; panel **(D)** is the trajectory of CDR; panel **(E)** is the trajectory of IQCODE, and panel **(F)** is the cumulative hazard of dementia incidence. MMSE, Mini-Mental Scale Estimation; MoCA, Montreal Cognitive Assessment; DRS, Mattis Dementia Rating Scale; CDR, Clinical Dementia Rating; IQCODE, Informant Questionnaire on Cognitive Decline in the Elderly; APOE, apolipoprotein E.

The declining trends in MMSE, MoCA, and DRS and the increasing trends in CDR and IQCODE were the lowest in the combination * ε4(−) group and the highest in the control * ε4(+) group after the patients were grouped based on telmisartan, rosuvastatin, and *APOE ε4* genotype. There were significant differences in the changing trends among these groups (all *P*_adjusted_ < 0.05; Figure 5). Considering the changes in MMSE, MoCA, DRS, CDR, and IQCODE scores from the baseline, we did not find significant differences between the rosuvastatin * ε4(−) and rosuvastatin * ε4(+) groups and between the combination * ε4(−) and combination * ε4(+) groups (all *P*_adjusted_ > 0.05; Supplementary Figure S4). The cumulative hazards of the incidences of dementia were the lowest in the combination * ε4(−) group and the highest in the control * ε4(+) group (Figure 5; Supplementary Table S1). There were interactions among telmisartan, rosuvastatin, and *APOE ε4* genotype on the changing trends in MMSE (*P*_adjusted_ = 0.029), MoCA (*P*_adjusted_ = 0.033), DRS (*P*_adjusted_ = 0.040), CDR (*P*_adjusted_ = 0.036), and IQCODE (*P*_adjusted_ = 0.031) and in the incidences of dementia (*P*_adjusted_ = 0.028) after adjustment for confounders.

### Changes in Blood Pressure, Lipids, and Fasting Plasma Glucose and Cumulative Hazards of Stroke Incidence

Supplementary Figures S5–S8 summarized the changes in blood pressure, lipids, and fasting plasma glucose and the cumulative hazards of stroke during the follow-up period in the patients after being grouped by different classification methods.

## Discussion

In this randomized clinical trial, we investigated the effects of antihypertensive and anti-lipidemic treatment on dementia, stroke incidence, and all-cause mortality in older hypertensive patients. Furthermore, the impact of *APOE* genotypes on the treatment was investigated in this study. The main findings were as follows: (1) both telmisartan and rosuvastatin alleviated the progression of cognitive impairment and reduced the risk of dementia; (2) telmisartan synergistically interacts with rosuvastatin to reduce the progression of cognitive impairment and the risk of dementia; (3) the hypertensive patients with *APOE ε4* allele had a higher risk of cognitive impairment progression and the dementia incidence than those without *APOE ε4* allele; and (4) rosuvastatin significantly alleviated the progression of cognitive impairment and the risks of dementia in patients with *APOE ε4* allele.

Although the argument on the effects of sartans and statins alleviating the progression of cognitive impairment and reducing the risk of dementia remains (Diener et al., 2008; Kehoe et al., 2009; Tsukuda et al., 2009; Power et al., 2015; Geifman et al., 2017; Ho and Nation, 2017; Zissimopoulos et al., 2017; Ahmed et al., 2018; Petek et al., 2018; Sinyavskaya et al., 2018), we found that both telmisartan and rosuvastatin significantly alleviated the progression of cognitive impairment and reduced the risk of dementia even after adjustment for confounders including the level of education and the status of hydrochlorothiazide administration. A previous study indicated that telmisartan performed slightly better than placebo on attenuating the progression of cognitive impairment and the risk of dementia (Zhang et al., 2019).

In a case-control and retrospective cohort study, the use of statins was associated with a lower prevalence of dementia and improved MMSE scores compared to patients who did not receive statin treatment (Hajjar et al., 2002). A previous study (Ji et al., 2018) showed that the risk of white matter hyperintensity progression was lower in the rosuvastatin than in the placebo group, which could underlie the lower risk of dementia and cognitive impairment progression in the former patients.

In this study, we observed that telmisartan synergistically interacted with rosuvastatin to reduce the progression of cognitive impairment and the risks of dementia. This result may be due to the fact that telmisartan mainly lowers blood pressure whereas rosuvastatin mainly lowers plasma lipid. As is known, hypertension is often accompanied by dyslipidemia. Another reason might be that telmisartan and rosuvastatin exhibit pleiotropic and protective effects on the cardio- and cerebrovascular systems including anti-inflammatory and antioxidant effects (Rizos et al., 2013; Liu et al., 2014), which is significant since inflammation and oxidative stress play important roles in dementia and cognitive impairment (Bulzacka et al., 2016; Liu et al., 2018).

Genotype frequencies can be used to evaluate the efficacy of health care practices. In this study, we observed that the risk of dementia in the patients with *APOE* ε4 allele was higher than those without *APOE* ε4 allele irrespective of treatment they received. We did not find any interaction between telmisartan and *APOE* ε4 allele. However, as expected, a significant interaction was found between rosuvastatin and *APOE* ε4 allele. The differences in the changes in global cognitive function from baseline were not significant between rosuvastatin * ε4(−) and rosuvastatin * ε4(+) groups and between combination * ε4(−) and combination * ε4(+) groups. A previous study showed that the risk factors for the incidence of small cerebral vessel diseases including white matter hyperintensities, lacunes, and microbleeds that were strongly associated with dementia and cognitive impairment in subjects with *APOE* ε4 allele were markedly mitigated by rosuvastatin (Ji et al., 2018). Meanwhile, *APOE* ε4 allele is closely associated with elevated levels of plasma lipids and has been demonstrated to increase the risk of dementia and cognitive impairment (Mahely and Huang, 1999; Reitz, 2012). Thus, the risks of dementia might be reduced by rosuvastain in subjects with *APOE* ε4 allele that has been regarded as a statin treatment threshold in clinical practice (Davies et al., 2011).

One of the major strengths of our study is that dementia was diagnosed using the combination of global cognitive function scale testing the patients themselves and based on the informant interview on cognitive decline. Second, the changes in blood pressure and lipids and the stroke incidence were included as confounders in analysis models. Third, the impact of *APOE* genotype on the treatment of telmisartan, rosuvastatin, and their combination was examined. The randomized, double-blind, placebo-controlled design and long-term follow-up might also be an advantage in this study.

On the other hand, our study also had several limitations. Firstly, hydrochlorothiazide was used as a basic medication in all patients. It is difficult to distinguish between these two groups in terms of differences in the incidence of dementia and cognitive impairment, although the status of hydrochlorothiazide administration was adjusted in the analysis models. Second, we did not consider the effects of ethnicity and gender. A previous meta-analysis showed that these factors do not alter the influence of the *APOE ε4* allele in dementia (Farrer et al., 1997), whereas others have reported that the effects of statins on Alzheimer’s disease patients varied according to race (Zissimopoulos et al., 2017). The differences in gender may affect the treatments of statins and sartans on cognitive impairment (Zissimopoulos et al., 2017; Ji et al., 2018). Third, a lower socioeconomic status was found to be closely associated with an increased risk of dementia and cognitive decline (Russ et al., 2013; Rusmaully et al., 2017). Socioeconomic background could bias the results of this study. Fourth, dementia was not subclassified into Alzheimer’s disease, senile dementia, and vascular dementia in this study. Lastly, we did not assess the effect of antiplatelet agents and antihyperglycemic medications as potential confounders on cognitive impairment and dementia in this study.

In conclusion, after over an average of 7 years’ follow-up, our findings indicate that the combination of telmisartan and rosuvastatin might be an effective prevention and/or treatment strategy for cognitive impairment and dementia in hypertensive patients, especially in those with *APOE ε4* allele. However, our results should be validated by additional studies which take into account the differences in ethnicity, socioeconomic background, and statin agents.

## Data Availability Statement

The datasets used and/or analyzed during the current study are available from the corresponding author on reasonable request.

## Ethics Statement

This study involving patients were reviewed and approved by the Research Ethics Committee of the Institute of Basic Medicine, Shandong Academy of Medical Sciences (registration number: 2007-12-09) on December 9, 2007. Each patient gave a written informed consent to participate in this study.

## Author Contributions

ZL, HZ, and YZ had full access to all the data in the study and had final responsibility for the decision to submit for publication and planned and initiated the trial. HZ, YL, and YD contributed to the management of data. ZL, WH, JW, and HZ contributed to the analysis and interpretation of data. WH, YL, and ZL contributed to the drafting of the manuscript. ZL, GG, and QC contributed to the critical revision of the manuscript for important intellectual content. All the authors contributed to the data collection. All authors interpreted data, critically reviewed the report, and approved the final version of the report.

## Conflict of Interest

The authors declare that the research was conducted in the absence of any commercial or financial relationships that could be construed as a potential conflict of interest.

## References

[B1] Ahmed H. A. Ishrat T. Pillai B. Fouda A. Y. Sayed M. A. Eldahshan W.. (2018). RAS modulation prevents progressive cognitive impairment after experimental stroke: a randomized, blinded preclinical trial. J. Neuroinflammation 15:229. 10.1186/s12974-018-1262-x 30103772 PMC6090822

[B2] Albert M. S. Dekosky S. T. Dickson D. Dubois B. Feldman H. H. Fox N. C.. (2011). The diagnosis of mild cognitive impairment due to Alzhermer’s disease: recommendations from the National Institute on Aging-Alzheimer’s Association workgroups on diagnostic guidelines for Alzheimer’s disease. Alzheimers Dement. 7, 270–279. 10.1016/j.jalz.2011.03.008 21514249 PMC3312027

[B3] Arnett D. K. Blumenthal R. S. Albert M. A. Buroker A. B. Goldberger Z. D. Hahn E. J.. (2019). 2019 ACC/AHA guideline on the primary prevention of cardiovascular disease: a report of the american college of cardiology/american heart association task force on clinical practice guidelines. Circulation 140, e596–e646. 10.1161/CIR.0000000000000678 30879355 PMC7734661

[B4] Biessels G. J. Staekenborg S. Brunner E. Brayne C. Scheltens P. (2006). Risk of dementia in diabetes mellitus: a systematic review. Lancet Neurol. 5, 64–74. 10.1016/S1474-4422(05)70284-2 16361024

[B5] Bulzacka E. Boyer L. Schürhoff F. Godin O. Berna F. Brunel L.. (2016). Chronic peripheral inflammation is associated with cognitive impairment in schizophrenia: results from the multicentric FACE-SZ dataset. Schizophr. Bull. 42, 1290–1302. 10.1093/schbul/sbw029 27143795 PMC4988740

[B6] Chan A. S. Choi M. K. Salmon D. P. (2001). The effects of age, education, and gender on the Mattis Dementia Rating Scale performance of elderly Chinese and American individuals. J. Gerontol. B. Psychol. Sci. Soc. Sci. 56, P356–P363. 10.1093/geronb/56.6.p356 11682589

[B7] Cholesterol Treatment Trialists’ Collaboration. (2019). Efficacy and safety of statin therapy in older people: a meta-analysis of individual participant data from 28 randomised controlled trials. Lancet 393, 407–415. 10.1016/S0140-6736(18)31942-1 30712900 PMC6429627

[B8] Davies N. M. Windmeijer F. Martin R. M. Abdollahi M. R. Smith G. D. Lawlor D. A.. (2011). Use of genotype frequencies in medicated groups to investigate prescribing practice: APOE and statins as a proof of principle. Clin. Chem. 57, 502–510. 10.1373/clinchem.2010.156356 21228258

[B9] Delgado C. Araneda A. Behrens M. I. (2017). Validation of the Spanish-language version of the motreal cognitive assessment test in adults older than 60 years. Neurologia 34, 376–385. 10.1016/j.nrl.2017.01.013 28364958

[B10] Dergunov A. D. (2011). Apolipoprotein E genotype as a most significant predictor of lipid response at lipid-lowering therapy: mechanistic and clinical studies. Biomed. Pharmacother. 65, 597–603. 10.1016/j.biopha.2011.04.003 21705182

[B11] Diener H. C. Sacco R. L. Yusuf S. Cotton D. Ounpuu S. Lawton W. A.. (2008). Effects of aspirin plus extended-telease dipyridamole versus clopidogrel and telmisartan on disability and cognitive function after recurrent stroke in patients with ischaemic stroke in the Prevention Regimen for Effectively Avoiding Second Strokes (PRoFESS) trial: a double-blind, active and placebo-controlled study. Lancet Neurol. 7, 875–884. 10.1016/S1474-4422(08)70198-4 18757238 PMC2772657

[B12] Duan D. Dong Y. Zhang H. Zhao Y. Diao Y. Cui Y.. (2017). Empty-nest-related psychological distress is associated with progression of brain white matter lesions and cognitive impairment in the elderly. Sci. Rep. 7:43816. 10.1038/srep43816 28256594 PMC5335556

[B13] Farao G. Iadecola C. (2013). Hypertension: a harbinger of stroke and dementia. Hypertension 62, 810–817. 10.1161/hypertensionaha.113.01063 23980072 PMC3847558

[B14] Farrer L. A. Cupples L. A. Haines J. L. Hyman B. Kukull W. A. Mayeux R.. (1997). Effects of age, sex, and ethnicity on the association between apoliporotein E genotype and Alzheimer disease. A meta-analysis. APOE and Alzheimer Disease Meta Analysis Consortium. JAMA 278, 1349–1356. 10.1001/jama.278.16.1349 9343467

[B15] Fuh J. L. Teng E. L. Lin K. N. Larson E. B. Wang S. J. Liu C. Y.. (1995). The Informant Questionniare on Cognitive Decline in the Elderly (IQCODE) as a screening tool for dementia for a predominantly illiterate Chinese population. Neurology 45, 92–96. 10.1212/wnl.45.1.92 7824143

[B16] Gavett R. A. Dunn J. E. Stoddard A. Harty B. Weintraub S. (2011). The Cognitive Change in Women study (CCW): informant ratings of cognitive change but not selft ratings are associated with neuropsychological performance over three years. Alzheimer. Dis. Assoc. Disord. 25, 305–311. 10.1097/wad.0b013e31820d8652 22086219 PMC3220881

[B17] Geifman N. Brinton R. D. Kennedy R. E. Schneider L. S. Butte A. J. (2017). Evidence for benefit of statins to modify cognitive decline and risk in Alzheimer’s disease. Alzheimers Res. Ther. 9:10. 10.1186/s13195-017-0237-y 28212683 PMC5316146

[B18] Gorelick P. B. Scuteri A. Black S. E. Decarli C. Greenberg S. M. Iadecola C.. (2011). Vascular contributions to cognitive impairment and dementia: a statement for healthcare professionals from the American Heart Association/American Stroke Association. Stroke 42, 2672–2713. 10.1161/STR.0b013e3182299496 21778438 PMC3778669

[B19] Hajjar I. Schumpert J. Hirth V. Wieland D. Eleazer G. P. (2002). The impact of the use of statins on the prevalence of dementia and the progression of cognitive impairment. J. Gerontol. A. Biol. Sci. Med. Sci. 57, M414–M418. 10.1093/gerona/57.7.m414 12084801

[B20] Harrison J. K. Stott D. J. McShane R. Noel-Storr A. H. Swann-Price R. S. Quinn T. J. (2016). Informant Questionnaire on Cognitive Decline in the Elderly (IQCODE) for the early diagnosis of dementia across a variety of healthcare settings. Cochrane Database Syst. Rev. 11:CD011333. 10.1002/14651858.cd011333.pub2 27869298 PMC6477966

[B21] Ho J. K. Nation D. A. Alzheimer’s Disease Neuroimaging Initiative. (2017). Memory is preserved in older adults taing AT1 receptor blockers. Alzheimers Res. Ther. 9:33. 10.1186/s13195-017-0255-9 28446207 PMC5405458

[B22] Ji T. Zhao Y. Wang J. Cui Y. Duan D. Chai Q.. (2018). Effect of low-dose statins and apolipoprotein E genotype on cerebral small vessel disease in older hypertensive patients: a subgroup analysis of a randomized clinical trial. J. Am. Med. Dir. Assoc. 19, 995.e4–1002.e4. 10.1016/j.jamda.2018.05.025 30006015

[B23] Jia J. Wang F. Wei C. Zhou A. Jia X. Li F.. (2014). The prevalence of dementia in urban and rural areas of China. Alzheimers Dement. 10, 1–9. 10.1016/j.jalz.2013.01.012 23871765

[B24] Jick H. Zornberg G. L. Jick S. S. Seshadri S. Drachman D. A. (2000). Statins and the risk of dementia. Lancet 356, 1627–1631. 10.1016/s0140-6736(00)03155-x 11089820

[B25] Jorm A. F. (1994). A short form of the Informant Questionnaire on Cognitive Decline in the Elderly (IQCODE): development and cross-validation. Psychol. Med. 24, 145–153. 10.1017/s003329170002691x 8208879

[B26] Jorm A. F. (2004). The Informant Questionnaire on cognitive decline in the elderly (IQCODE): a review. Int. Psychogeriatr. 16, 275–293. 10.1017/s1041610204000390 15559753

[B27] Jorm A. F. Jacomb P. A. (1989). The Informant Questionnaire on Cognitive Decline in the Elderly (IQCODE): socio-demographic correlates, reliability, validity and some norms. Psychol. Med. 19, 1015–1022. 10.1017/s0033291700005742 2594878

[B28] Kehoe P. Miners S. Love S. (2009). Angiotensins in Alzheimer’s disease-friend of foe? Trends Neurosci. 32, 619–628. 10.1016/j.tins.2009.07.006 19796831

[B31] Liu Z. Zhao Y. Wang X. Zhang H. Cui Y. Diao Y.. (2016). Low carotid artery wall shear stress is independently associated with brain white-matter hyperintensities and cognitive impairment in older patient. Atherosclerosis 247, 78–86. 10.1016/j.atherosclerosis.2016.02.003 26868512

[B30] Liu Z. Zhao Y. Wei F. Ye L. Lu F. Zhang H.. (2014). Treatment with telmisartan/rosuvastatin combination has a beneficial synergistic effect on ameliorating Th17/Treg functional imbalance in hypertensive patients with carotid atherosclerosis. Atherosclerosis 233, 291–299. 10.1016/j.atherosclerosis.2013.12.004 24495792

[B29] Liu W. Zhao Y. Zhang X. Ji J. (2018). Simvastatin ameliorates cognitive impairments *via* inhibition of oxidative stress-induced apoptosis of hippocampal cells through the ERK/AKT signaling pathway in a rat model of senile dementia. Mol. Med. Rep. 17, 1885–1892. 10.3892/mmr.2017.8098 29257256

[B32] Mahely R. W. Huang Y. (1999). Apolipoprotein E: from atherosclerosis to Alzheimer’s disease and beyond. Curr. Opin. Lipidol. 10, 207–217. 10.1097/00041433-199906000-00003 10431657

[B33] Molinuevo J. L. Gramunt N. Gispert J. D. Fauria K. Esteller M. Minguillon C.. (2016). The AlFA project: a research platform to identify early pathophysiological features of Alzheimer’s disease. Alzheimers Dement. 2, 82–92. 10.1016/j.trci.2016.02.003 29067295 PMC5644283

[B34] Morales J. M. Gonzalez-Montalvo J. I. Bermejo F. Del-Ser T. (1995). The screening of mild dementia with a shortened Spanish version of the “Informant Questionnaire on Cognitive Decline in the Elderly”. Alzheimer. Dis. Assoc. Disord. 9, 105–111. 10.1097/00002093-199509020-00008 7662322

[B35] Mortensen M. B. Nordestgaard B. G. (2018). Comparison of five major guidelines for statin use in primary prevention in a contemporary general population. Ann. Intern. Med. 168, 85–92. 10.7326/m17-0681 29297004

[B36] Narasimhalu K. Lee J. Auchus A. P. Chen C. P. (2008). Improving detection of dementia in Asian patients with low education: combining the Mini-mental State Examination and the Informant Questionnaire on Cognitive Decline in the Elderly. Dement. Geriatr. Gogn. Disord. 25, 17–22. 10.1159/000111128 18025825

[B37] Nazarian A. Arbeev K. G. Yashkin A. P. Kulminski A. M. (2019). Genetic heterogeneity of Alzheimer’s disease in subjects with and without hypertension. Geroscience 41, 137–154. 10.1007/s11357-019-00071-5 31055733 PMC6544706

[B38] Peng J. Lu F. Wang Z. Zhong M. Sun L. Hu N.. (2014). Excessive lowering of blood pressure is not beneficial for progression of brain white matter hyperintensive and cognitive impairment in elderly hypertensive patients: 4-year follow-up study. J. Am. Med. Dir. Assoc. 15, 904–910. 10.1016/j.jamda.2014.07.005 25239015

[B39] Petek B. Villa-Lopez M. Loera-Valencia R. Gerenu G. Winblad B. kramberger M. G.. (2018). Connecting the brain cholesterol and rennin-angiotensin systems: potential role of statins and RAS-modifying medications in dementia. J. Intern. Med. 284, 620–642. 10.1111/joim.12838 30264910

[B40] Power M. C. Weuve J. Sharrett A. R. Blacker D. Gottesman R. F. (2015). Statins, cognition and dementia - systematic review and methodological commentary. Nat. Rev. Neurol. 11, 220–229. 10.1038/nrneurol.2015.35 25799928 PMC4458855

[B41] Prince M. Bryce R. Albanese E. Wimo A. Riberiro W. Ferri C. P. (2013). The global prevalence of dementia: a systematic review and metaanalysis. Alzheimers Dement. 9, 63–75. 10.1016/j.jalz.2012.11.007 23305823

[B42] Reitz C. (2012). Dyslipidemia and dementia: current epidemiology, genetic evidence, and mechanisms behind the associations. J. Alzheimers Dis. 30, 127–145. 10.3233/jad-2011-110599 21965313 PMC3689537

[B43] Rizos C. V. Liberopoulos E. N. Tellis C. C. Tselepis A. D. Elisaf M. S. (2013). The effect of combining rosuvastatin with sartans of different peroxisome proliferator receptor-γ activating capacity on plasma 8-isoprostane prostaglandin F2α levels. Arch. Med. Sci. 9, 172–176. 10.5114/aoms.2013.33357 23515108 PMC3598137

[B44] Rodrigues E. G. Dodge H. H. Birzescu M. A. Stoehr G. P. Ganguli M. (2002). Use of lipid-lowering drugs in older adults with and without dementia: a community-based epidemiological study. J. Am. Geriatr. Soc. 50, 1852–1856. 10.1046/j.1532-5415.2002.50515.x 12410906

[B45] Rusmaully J. Dugravot A. Moatti J. P. Marmot M. G. Elbaz A. Kivimaki M.. (2017). Contribution of cognitive performance and cognitive decline to associations between socioeconomic factors and dementia: a cohort study. PloS Med. 14:e1002334. 10.1371/journal.pmed.1002334 28650972 PMC5484463

[B46] Russ T. C. Stamatakis E. Hamer M. Starr J. M. Kivimäki M. Batty G. D. (2013). Socioeconomic status as a risk factor for dementia death: individual participant meta-analysis of 86 508 men and women from the UK. Br. J. Psychiatry 203, 10–17. 10.1192/bjp.bp.112.119479 23818534 PMC3696876

[B47] Sabia S. Dugravot A. Dartigues J. F. Abell J. Elbaz A. Kivimäki M.. (2017). Physical activity, cognitive decline and risk of dementia: 28 year follow-up of Whitehall II cohort study. BMJ 357:j2709. 10.1136/bmj.j2709 28642251 PMC5480222

[B48] Shah H. Albanese E. Duggan C. Rudan I. Langa K. M. Carrillo M. C.. (2016). Research priorities to reduce the global burden of dementia by 2025. Lancet Neurol. 15, 1285–1294. 10.1016/s1474-4422(16)30235-6 27751558

[B49] Sinyavskaya L. Gauthier S. Renoux C. Dell’Aniello S. Suissa S. Brassard P. (2018). Comparative effect of statins on the risk of incident Alzheimer disease. Neurology 90, e179–e187. 10.1212/wnl.0000000000004818 29247073

[B50] Sörös P. Whitehead S. Spence J. D. Hachinski V. (2013). Antihypertensive treatment can prevent stroke and cognitive decline. Nat. Rev. Neurol. 9, 174–178. 10.1038/nrneurol.2012.255 23247612

[B51] Tsukuda K. Mogi M. Iwanami J. Min L. J. Sakata A. Jing F.. (2009). Cognitive deficit in amyloid-β-injected mice was improved by pretreatment with a low dose of telmisartan partly because of peroxisome proliferators-activated receptor-γ activation. Hypertension 54, 782–787. 10.1161/hypertensionaha.109.136879 19635982

[B52] US Preventive Services Task Force Bibbins-Domingo K. Grossman D. C. Curry S. J. Davidson K. W. Epling J. W. Jr.. (2016). Statin use for the primary prevention of cardiovascular disease in adults: US Preventive Services Task Force Recommendation Statement. JAMA 316, 1997–2007. 10.1001/jama.2016.15450 27838723

[B53] Van Middelaar T. van Vught L. A. van Gool W. A. Simons E. M. van den Born B. H. Moll van Charante E. P.. (2018). Blood pressure-lowering interventions to prevent dementia: a systematic review and meta-analysis. J. Hypertens. 36, 1780–1787. 10.1097/hjh.0000000000001829 29927845

[B54] Yue W. Wang X. D. Shi Z. Wang Y. Liu S. Liu S.. (2016). The prevalence of dementia with lewy bodies in a rural area of China. Parkinsonism Relat. Disord. 29, 72–77. 10.1016/j.parkreldis.2016.05.022 27264344

[B55] Zhang H. Cui Y. Zhao Y. Dong Y. Duan D. Wang J.. (2019). Effects of sartans and low-dose statins on cerebral white matter hyperintensities and cognitive function in older patients with hypertension: a randomized, double-blind and placebo-controlled clinical trial. Hypertens. Res. 42, 717–729. 10.1038/s41440-018-0165-7 30552406

[B56] Zissimopoulos J. M. Barthold D. Brinton R. D. Joyce G. (2017). Sex and race differences in the association between statin use and the incidence of Alzheimer disease. JAMA Neurol. 74, 225–232. 10.1001/jamaneurol.2016.3783 27942728 PMC5646357

