# Supplementary material for: Telmisartan and Rosuvastatin Synergistically Ameliorate Dementia and Cognitive Impairment in Older Hypertensive Patients With Apolipoprotein E Genotype
Source: Front Aging Neurosci. 2020 Jun 9;12:154. doi: 10.3389/fnagi.2020.00154 (PMC7296075; doi:10.3389/fnagi.2020.00154)
Supplement: Supplementary file 1 [file Data_Sheet_1.pdf]

# Telmisartan and rosuvastatin synergistically ameliorating dementia and cognitive impairment in older hypertensive patients with apolipoprotein E genotype

Wenjing Hu,[1,#] Ying Li,[2,#] Yingxin Zhao,[1] Yuanli Dong,[3] Yi Cui,[4] Shangwen Sun,[1] Gary Gong,[5] Hua Zhang,[1] Qiang Chai,[1] Juan Wang,[6*] Zhendong Liu,[1,*]

**Supplementary Table 1  The risks of the incidence of dementia**

| | HR | 95 % CI | *P* value |
|---|---|---|---|
| Grouped by telmisartan and rosuvastatin | | | |
| Combination group | Ref. | | |
| Rosuvastatin group | 1.904 | 1.137 to 3.188 | 0.014 |
| Telmisartan group | 2.064 | 1.241 to 3.433 | 0.005 |
| Control group | 3.835 | 2.835 to 6.137 | <0.001 |
| Grouped by telmisartan and APOE genotype | | | |
| Telmisartan(+)**ε4*(-) group | Ref. | | |
| Telmisartan(+)**ε4*(+) group | 1.339 | 0.785 to 2.286 | 0.284 |
| Telmisartan(-)**ε4*(-) group | 1.838 | 1.273 to 2.654 | 0.001 |
| Telmisartan(-)**ε4*(+) group | 2.617 | 1.691 to 4.050 | <0.001 |
| Grouped by rosuvastatin and APOE genotype | | | |
| Rosuvastatin(+)**ε4*(-) group | Ref. | | |
| Rosuvastatin(+)**ε4*(+)group | 1.276 | 0.737 to 2.208 | 0.384 |
| Rosuvastatin(-)**ε4*(-) group | 1.948 | 1.345 to 2.822 | <0.001 |
| Rosuvastatin(-)**ε4*(+) group | 2.895 | 1.869 to 4.486 | <0.001 |
| Grouped by telmisartan, rosuvastatin, and APOE genotype | | | |
| Combination**ε4*(-) group | Ref. | | |
| Combination**ε4*(+) group | 1.461 | 0.601 to 3.552 | 0.403 |
| Rosuvastatin**ε4*(-) group | 2.035 | 1.101 to 3.762 | 0.023 |
| Rosuvastatin**ε4*(+) group | 2.319 | 1.076 to 4.999 | 0.032 |
| Telmisartan**ε4*(-) group | 2.151 | 1.173 to 3.948 | 0.013 |
| Telmisartan**ε4*(+) group | 2.706 | 1.279 to 5.721 | 0.009 |
| Control**ε4*(-) group | 3.682 | 2.087 to 6.498 | <0.001 |
| Control**ε4*(+) group | 5.967 | 3.182 to 11.190 | <0.001 |

**Supplementary Figure legends:**

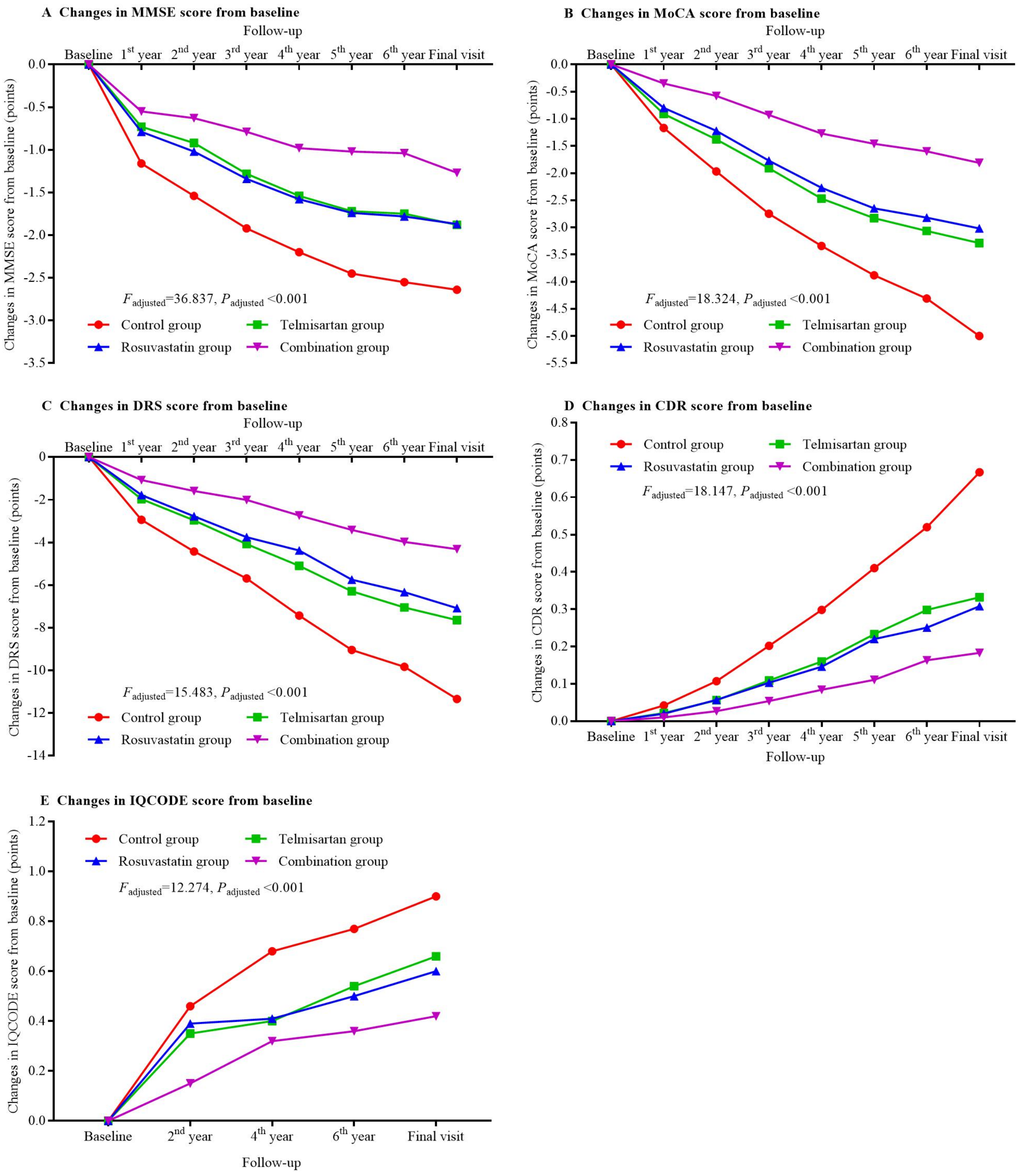


**Supplementary Fig. 1 Changes in global cognitive function and IQCODE from baseline at every follow-up visit in the patients grouped by telmisartan and rosuvastatin administration.** A is the changes in MMSE; B is the changes in MoCA; C is the changes in DRS; D is the changes in CDR; E is the changes in IQCODE. MMSE indicates Mini-Mental Scale Estimation; MoCA, Montreal Cognitive Assessment; DRS, Mattis Dementia Rating Scale; CDR, Clinical Dementia Rating; IQCODE, Informant Questionnaire on Cognitive Decline in the Elderly.

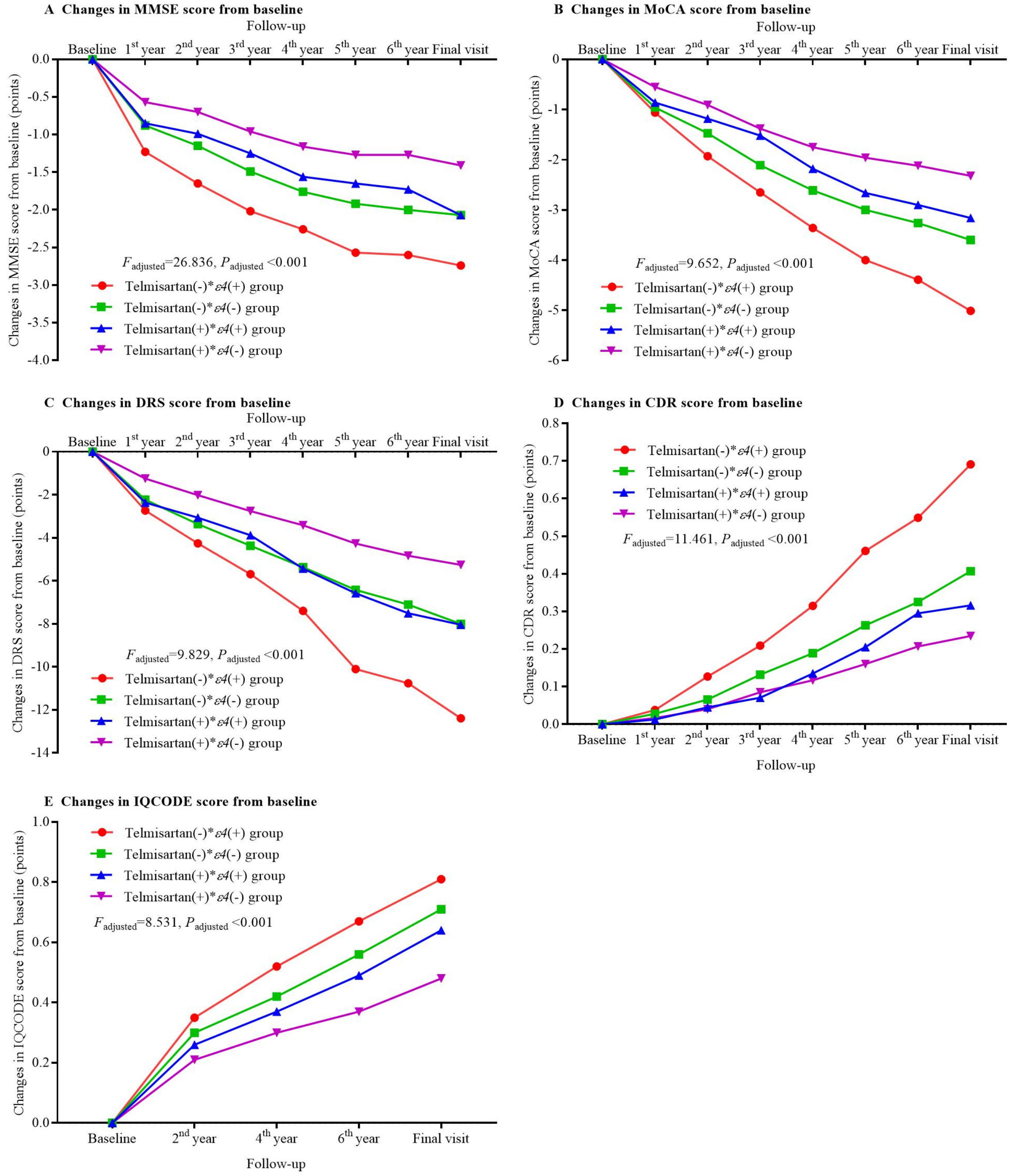


**Supplementary Fig. 2 Changes in global cognitive function and IQCODE from baseline at every follow-up visit in the patients grouped by telmisartan administration and *APOE* genotype.** A is the changes in MMSE; B is the changes in MoCA; C is the changes in DRS; D is the changes in CDR; E is the changes in IQCODE. MMSE indicates Mini-Mental Scale Estimation; MoCA, Montreal Cognitive Assessment; DRS, Mattis Dementia Rating Scale; CDR, Clinical Dementia Rating; IQCODE, Informant Questionnaire on Cognitive Decline in the Elderly.

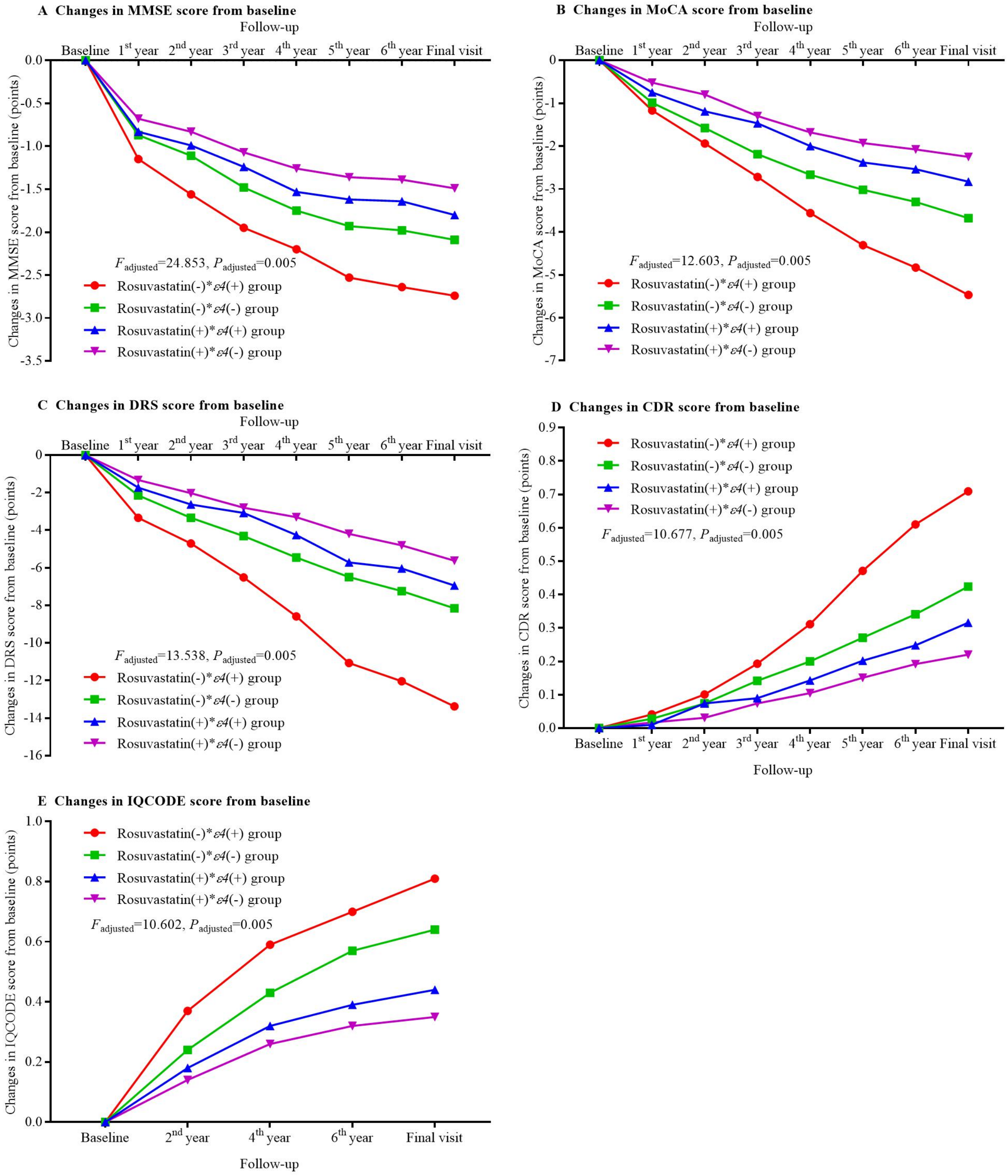


**Supplementary Fig. 3 Changes in global cognitive function and IQCODE from baseline at every follow-up visit in the patients grouped by rosuvastatin administration and *APOE* genotype.** A is the changes in MMSE; B is the changes in MoCA; C is the changes in DRS; D is the changes in CDR; E is the changes in IQCODE. MMSE indicates Mini-Mental Scale Estimation; MoCA, Montreal Cognitive Assessment; DRS, Mattis Dementia Rating Scale; CDR, Clinical Dementia Rating; IQCODE, Informant Questionnaire on Cognitive Decline in the Elderly.

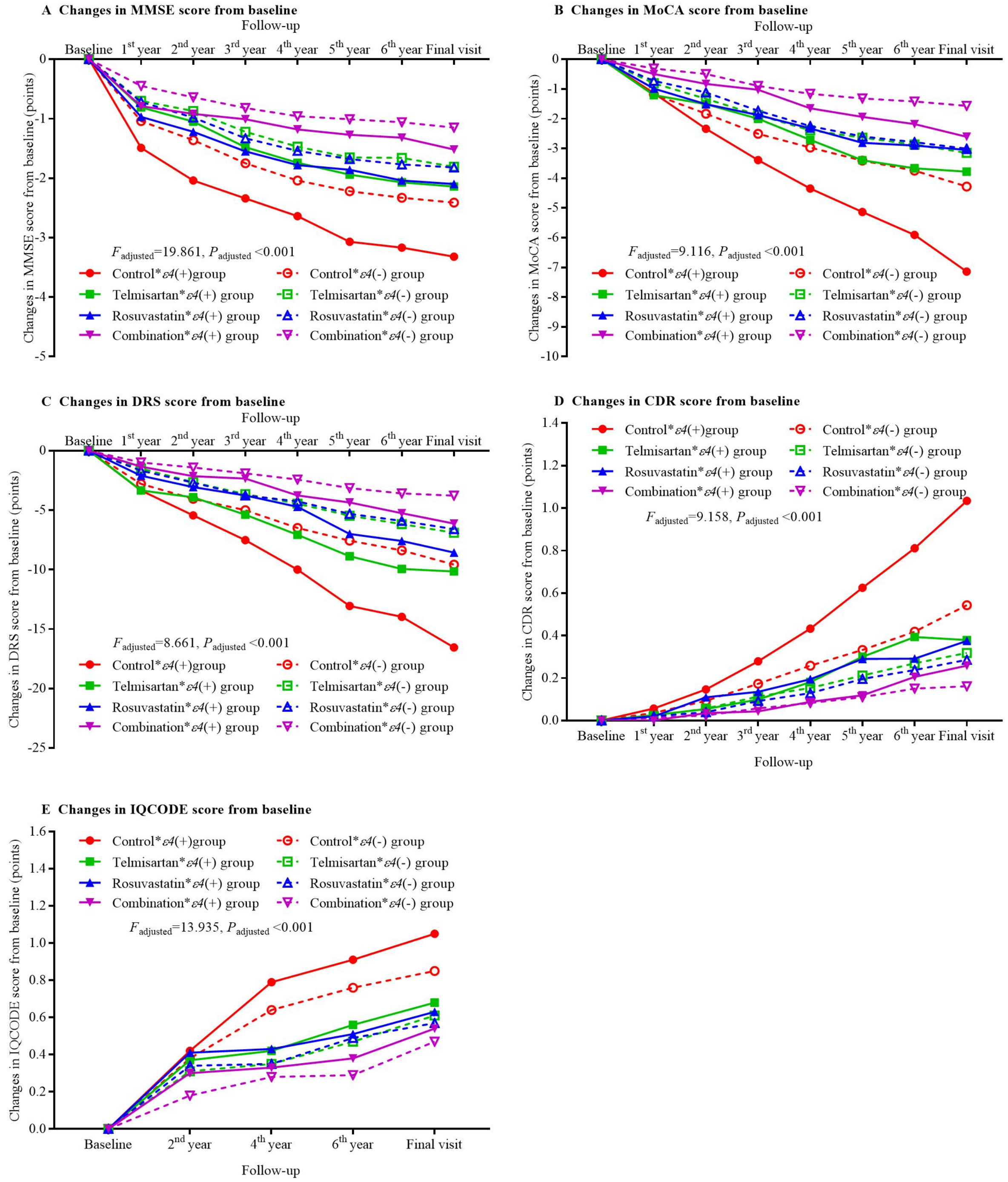


**Supplementary Fig. 4 Changes in global cognitive function and IQCODE from baseline at every follow-up visit in the patients grouped by telmisartan, rosuvastatin, and *APOE* genotype.** A is the changes in MMSE; B is the changes in MoCA; C is the changes in DRS; D is the changes in CDR; E is the changes in IQCODE. MMSE indicates Mini-Mental Scale Estimation; MoCA, Montreal Cognitive Assessment; DRS, Mattis Dementia Rating Scale; CDR, Clinical Dementia Rating; IQCODE, Informant Questionnaire on Cognitive Decline in the Elderly.

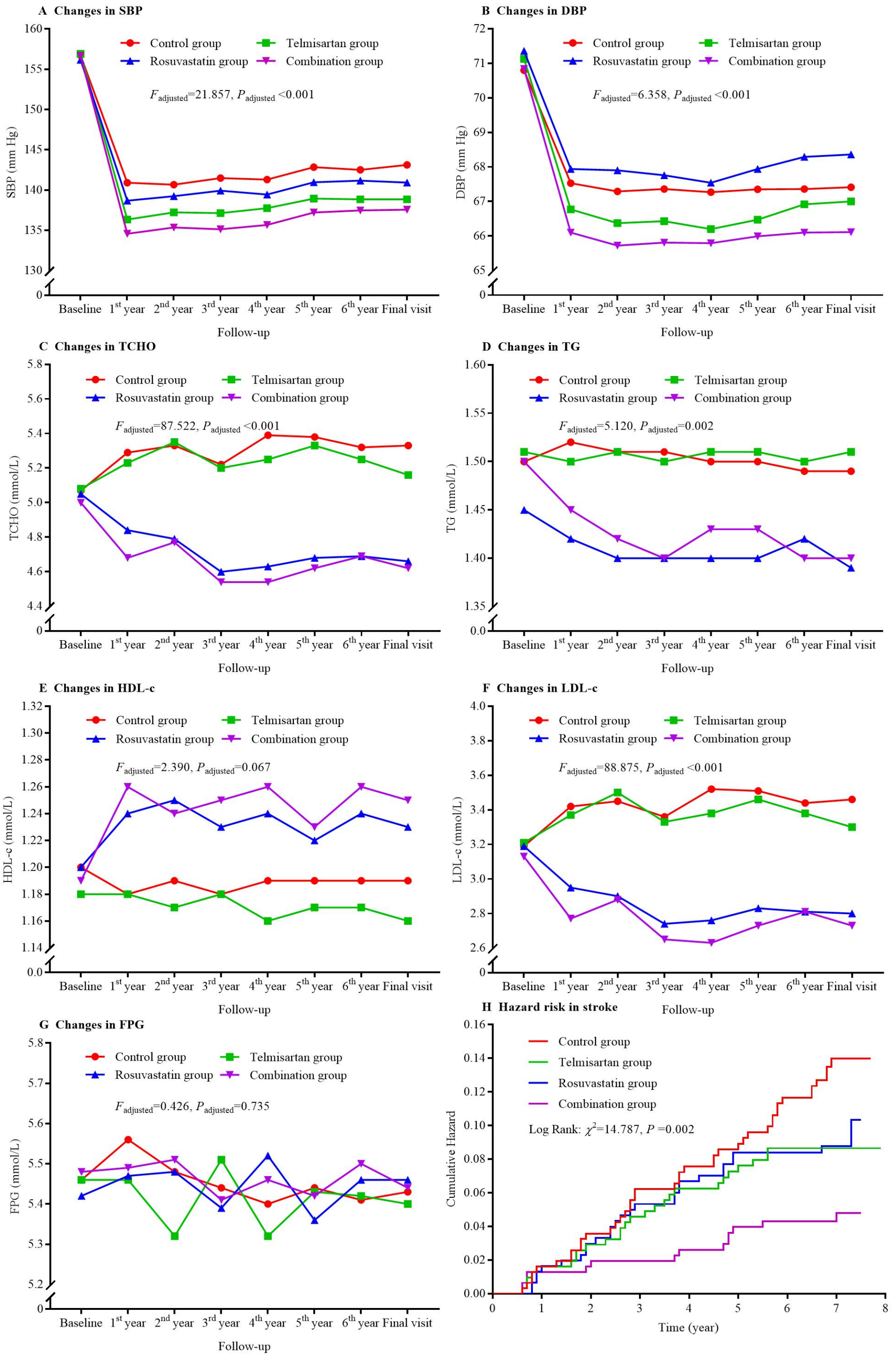


**Supplementary Fig. 5 Trajectory of blood pressure, lipids, and fasting plasma glucose and cumulative hazards of stroke incidence during the follow-up period in the patients grouped by telmisartan and rosuvastatin administration.** A is the trajectory of SBP; B is the trajectory of DBP; C is the trajectory of TCHO; D is the trajectory of TG; E is the trajectory of HDL-c; F is the trajectory of LDL-c; G is the trajectory of FPG; H is the cumulative hazard of stroke incidence. SBP indicates systolic blood pressure; DBP, diastolic blood pressure; TCHO, total cholesterol; TG, triglycerides; HDL-c, high-density lipoprotein cholesterol; LDL-c, low-density lipoprotein cholesterol; FPG, fasting plasma glucose.

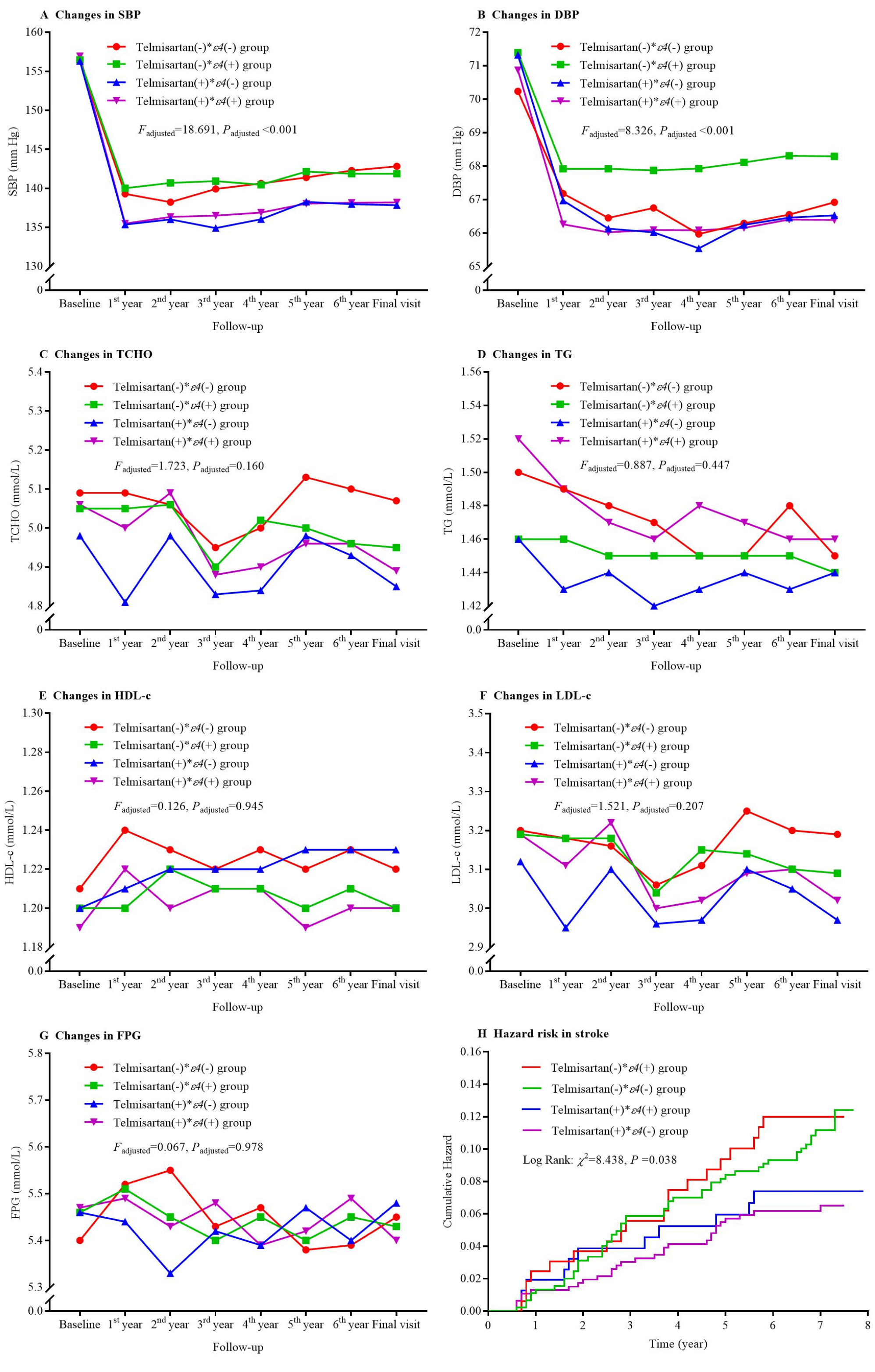


**Supplementary Fig. 6 Trajectory of blood pressure, lipids, and fasting plasma glucose and cumulative hazards of stroke incidence during the follow-up period in the patients grouped by telmisartan administration and *APOE* genotype.** A is is the trajectory of SBP; B is the trajectory of DBP; C is the trajectory of TCHO; D is the trajectory of TG; E is the trajectory of HDL-c; F is the trajectory of LDL-c; G is the trajectory of FPG; H is the

cumulative hazard of stroke incidence. SBP indicates systolic blood pressure; DBP, diastolic blood pressure; TCHO, total cholesterol; TG, triglycerides; HDL-c, high-density lipoprotein cholesterol; LDL-c, low-density lipoprotein cholesterol; FPG, fasting plasma glucose.

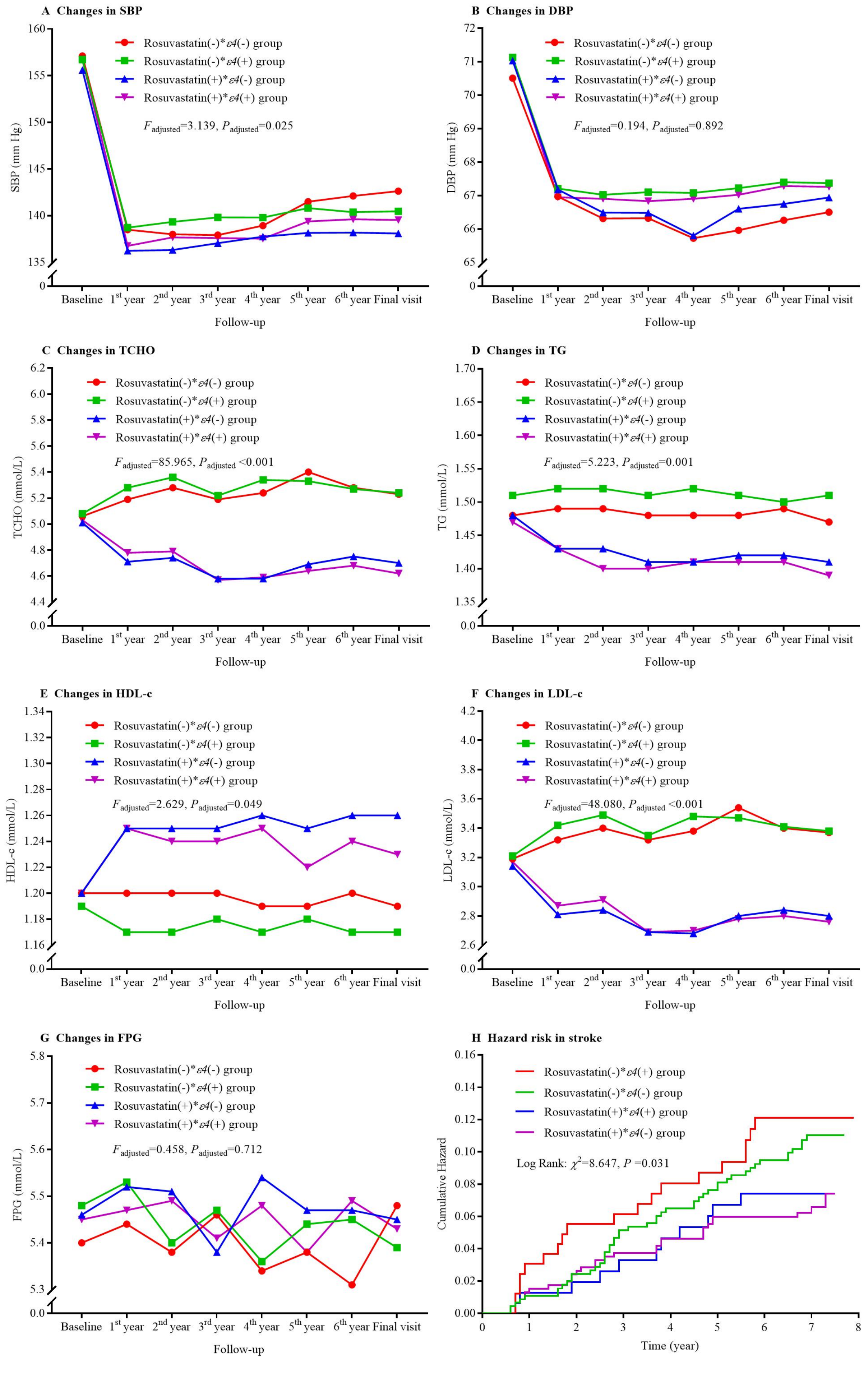


**Supplementary Fig. 7 Trajectory of blood pressure, lipids, and fasting plasma glucose and cumulative hazards of stroke incidence during the follow-up period in the patients**

**grouped by rosuvastatin administration and *APOE* genotype.** A is  is the trajectory of SBP; B is the trajectory of DBP; C is the trajectory of TCHO; D is the trajectory of TG; E is the trajectory of HDL-c; F is the trajectory of LDL-c; G is the trajectory of FPG; H is the cumulative hazard of stroke incidence. SBP indicates systolic blood pressure; DBP, diastolic blood pressure; TCHO, total cholesterol; TG, triglycerides; HDL-c, high-density lipoprotein cholesterol; LDL-c, low-density lipoprotein cholesterol; FPG, fasting plasma glucose.

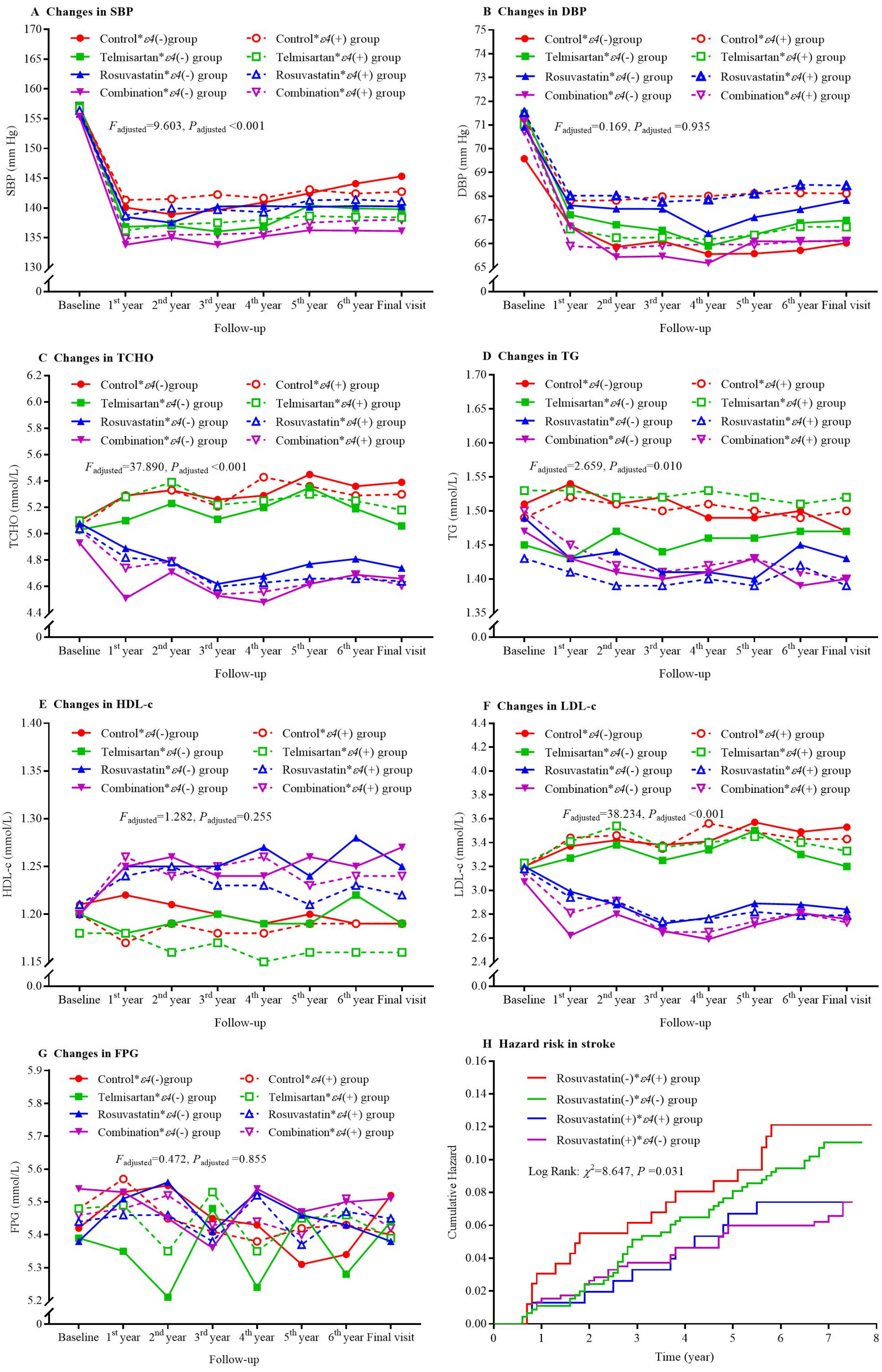


**Supplementary Fig. 8 Trajectory of blood pressure, lipids, and fasting plasma glucose and cumulative hazards of stroke incidence during the follow-up period in the patients**

**grouped by telmisartan, rosuvastatin, and *APOE* genotype.** A is is the trajectory of SBP; B is the trajectory of DBP; C is the trajectory of TCHO; D is the trajectory of TG; E is the trajectory of HDL-c; F is the trajectory of LDL-c; G is the trajectory of FPG; H is the cumulative hazard of stroke incidence. SBP indicates systolic blood pressure; DBP, diastolic blood pressure; TCHO, total cholesterol; TG, triglycerides; HDL-c, high-density lipoprotein cholesterol; LDL-c, low-density lipoprotein cholesterol; FPG, fasting plasma glucose.
